# Experimental and Theoretical Studies of Dissociative Electron Attachment to Metabolites Oxaloacetic and Citric Acids

**DOI:** 10.3390/ijms22147676

**Published:** 2021-07-18

**Authors:** Janina Kopyra, Paulina Wierzbicka, Adrian Tulwin, Guillaume Thiam, Ilko Bald, Franck Rabilloud, Hassan Abdoul-Carime

**Affiliations:** 1 Faculty of Exact and Natural Sciences, Siedlce University of Natural Sciences and Humanities, 3 Maja 54, 08-110 Siedlce, Poland; paulina.wierzbicka@uph.edu.pl (P.W.); adrian.tul@interia.pl (A.T.); 2 Université de Lyon, Université Claude Bernard Lyon 1, CNRS, Institut Lumière Matière, UMR5306, F-69622 Villeurbanne, France; guillaume.thiam@univ-lyon1.fr (G.T.); franck.rabilloud@univ-lyon1.fr (F.R.); 3 Institute of Chemistry, University of Potsdam, Karl-Liebknecht-Str. 24-25, 14476 Potsdam, Germany; 4 Institut de Physique des 2 Infinis, Université Lyon 1, Université de Lyon, CNRS/IN2P3, UMR5822, F-69003 Lyon, France; hcarime@ipnl.in2p3.fr

**Keywords:** dissociative electron attachment, negative ions, oxaloacetic acid, citric acid, mass spectrometry

## Abstract

In this contribution the dissociative electron attachment to metabolites found in aerobic organisms, namely oxaloacetic and citric acids, was studied both experimentally by means of a crossed-beam setup and theoretically through density functional theory calculations. Prominent negative ion resonances from both compounds are observed peaking below 0.5 eV resulting in intense formation of fragment anions associated with a decomposition of the carboxyl groups. In addition, resonances at higher energies (3–9 eV) are observed exclusively from the decomposition of the oxaloacetic acid. These fragments are generated with considerably smaller intensities. The striking findings of our calculations indicate the different mechanism by which the near 0 eV electron is trapped by the precursor molecule to form the transitory negative ion prior to dissociation. For the oxaloacetic acid, the transitory anion arises from the capture of the electron directly into some valence states, while, for the citric acid, dipole- or multipole-bound states mediate the transition into the valence states. What is also of high importance is that both compounds while undergoing DEA reactions generate highly reactive neutral species that can lead to severe cell damage in a biological environment.

## 1. Introduction

**Figure 1:**
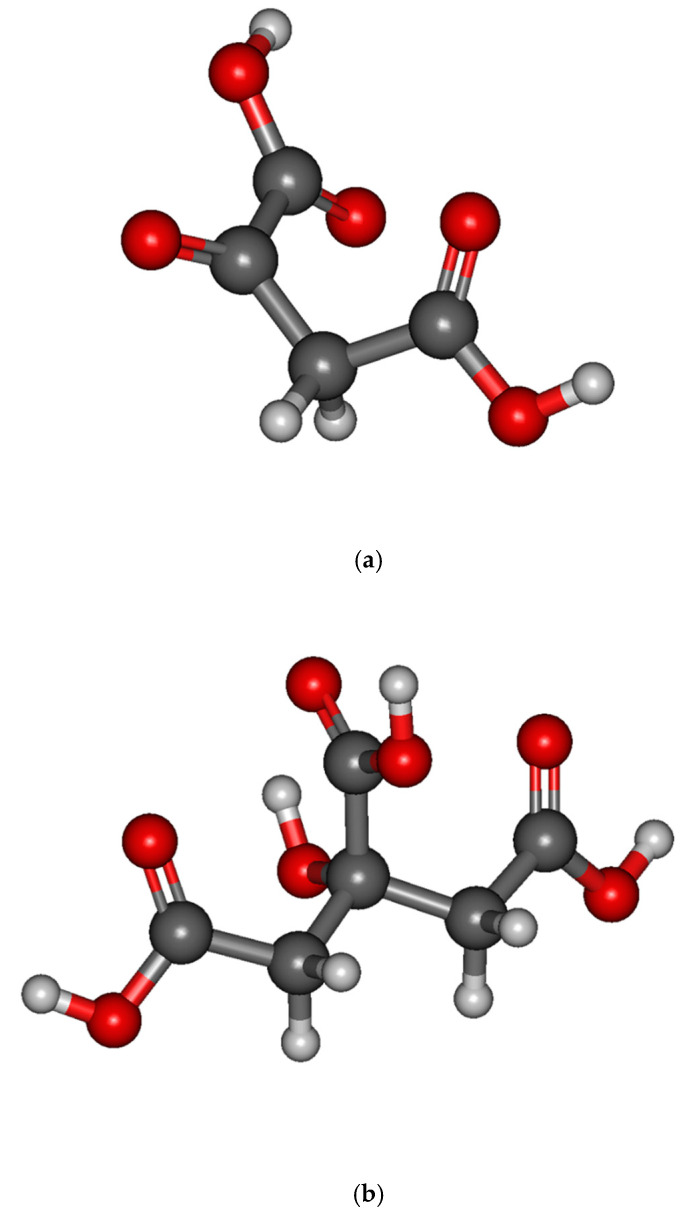
Optimized structure of the (**a**) neutral cis-enol oxaloacetic acid, (**b**) citric acid. The molecules were optimized at the ωB97x/cc-pvtz level of theory at 0 K.

Oxaloacetic acid (OAA, C_4_H_4_O_5_, m/z 132) and citric acid (CA, C_6_H_8_O_7_, m/z 192) are important metabolites found in aerobic organisms. Within the citrate (or Krebs) cycle, citrate is decomposed into oxaloacetate to release chemically stored energy. OAA possesses two carboxyl groups and a carbonyl group, while CA possesses three carboxyl groups and a hydroxyl group (Figure 1). The presence of these oxygen-rich functional groups is key to their electron-accepting properties, which are pivotal for the electron transfer and redox reactions taking place within the metabolism in the Krebs cycle. Although the electron transfer chains established in the organism are well-balanced, the delicate equilibria might be disturbed by the presence of “free” electrons generated, for example, by ionizing radiation through water radiolysis [1,2,3].

The last two decades have seen the implication of low energy electrons in various application fields as diverse as radiation chemotherapy, chemistry or nano-lithography [4,5,6,7,8]. In the irradiated material, the primary energetic particles or radiation generate a very large number of ballistic secondary electrons with an energy distribution below 10 eV [9]. These slow particles are now known to be able to induce efficient fragmentation to molecules as large as DNA [10]. Previous studies have shown that DNA is not the only critical target in biological radiation damage. In addition to DNA a decomposition of pivotal metabolites such as CA and OAA can also lead to dysfunction of the metabolism and ultimately to cell deaths. Identification of such critical damage pathways could trigger the development of more targeted therapies or radiation protection strategies, e.g., development of new radiosensitizers [11,12].

In addition to its biological role, CA is used as a reducing agent for nanoparticle synthesis and citrate serves as a stabilizing agent for example for gold nanoparticles. Gold nanoparticles are interesting due to their optical properties associated with the excitation of their surface plasmon resonance. Recent studies have shown that this excitation can lead to the formation of hot electrons within the nanoparticles, which can be transferred to adsorbed molecules [13,14] such as the stabilizing citrate molecules. Indeed, it was demonstrated that irradiation of citrate stabilized gold nanoparticles with visible light can lead to decomposition of the citrate [15]. In this context it is very important to characterize the interaction of citrate or the protonated form of citrate, i.e., CA, with low-energy electrons. Nanoparticles are also suggested as radiosensitizers in radiation therapy because they increase the local dose of LEEs upon irradiation [16]. In order to assess their mode of action, it is also important to characterize the interaction of LEEs with the stabilizing coating of nanoparticles.

In this work, we demonstrate that both oxaloacetic and citric acid are highly reactive towards electrons with very low energy, i.e., close to 0 eV. However, the involved processes at these energies differ according to the nature of the acids. Additionally, we observe the formation of highly reactive neutral species that can lead to severe cell damage in a biological environment.

## 2. Methodology

### 2.1. Experimental Procedure

The interaction of electrons with OAA and CA was studied by means of electron-molecular crossed-beam setup consisting of a cathode being a source of electrons, an oven, and a quadrupole mass analyzer (QMS) [17]. All elements of the experimental setup are located in an ultra-high vacuum chamber with a base pressure of 10^−9^ mbar. The working pressure was three (6.5 × 10^−6^ mbar) or two (1.6–2 × 10^−7^ mbar) orders of magnitude higher with respect to the base pressure for OAA and CA, respectively. A quasi-monoenergetic electron beam is generated from a trochoidal electron monochromator (FWHM of about 200 meV, electron current of 15-20 nA) and perpendicularly crosses with an effusive molecular beam of OAA or CA. The molecular beam emanates from the container containing the sample of the investigated compounds (Sigma Aldrich) heated by halogen bulbs located around the spectrometer. CA decomposes at temperatures above 434 K, as it has been shown by thermogravimetry and differential scanning calorimetry measurements [18]. OAA can adopt two conformations, the cis-enol and the trans-enol forms. The melting point of the cis-enol form is 425 K, and that of the trans-enol forms is higher, i.e., 457 K [19]. Since the experiments were undertaken at 400 K (for CA) and 379 K (for OAA), the electrons are likely interacting with unaltered intact molecules. OAA is likely to be produced mainly in the cis-enol structure. The negative ions formed from the integration of the electrons with the investigated compounds are extracted from the reaction zone by small draw-out-field (<1.0 Vcm^−1^), analyzed by the QMA and detected by single pulse counting technique. In the present experiment we use SF_6_ as a reference gas to calibrate the energy scale since it produces a near 0 eV [SF_6_]^−^ resonance. It should be noted that the experiments are done without the presence of the reference gas to avoid unwanted reactions of dissociative electron transfer with the target compounds [20].

### 2.2. Theoretical Method

Calculations were performed in the framework of the density-functional theory (DFT) using the Gaussian16 suite of programs [21]. We used the long-range-corrected hybrid density functional ωB97x [22] together with the Gaussian atomic basis set cc-pvtz and aug-cc-pvtz [23].

Two structures were optimized for the neutral OAA corresponding to the cis-enol form (Figure 1a) and the trans-enol (Figure S1), the former being more stable than the second one by 0.05 eV. The vertical electron affinity (VEA) defined as the energy difference between the neutral and lowest anion state, each in the optimized structure of the neutral molecule, is calculated at ωB97x/aug-cc-pvtz level. It is found to be positive by 0.20 eV (cis-enol) and 0.50 eV (trans-enol). The dipole moment for each structure is calculated to be 1.87 D and 2.25 D, respectively. The average quadrupole moment and the isotropic polarizability are calculated to be −49.08 D. Å and 9.43 Å^3^ for the cis-enol configuration and −49.80 D.Å and 9.57 Å^3^ for trans-enol configuration. The structure of the neutral CA is shown in Figure 1b. In contrast to OAA, the VEA of CA is calculated to be negative (−1.52 eV). The average dipole and quadrupole moments and isotropic polarizability are estimated to be 3.33 D, −71.12 D. Å and 14.08 Å^3^, respectively.

The prediction of resonant attachment energies can be investigated with approaches ranging from empirical methods to high-level ab initio calculation coupled with the stabilization method [24]. Here, we use an alternative, and less cumbersome, methodology to the stabilization method. The attachment energies of the incoming electron are calculated as the sum of the VEA and the excitation energies of the anion computed at TDDFT level (time-dependent DFT). For describing the valence-type excitations, TDDFT calculations are performed at ωB97x/cc-pvtz level, where the use of the relatively small basis set cc-pvtz (without any diffuse function) serves as a confinement for transitory states and is well suited to describe valence-type excitations [24]. However, for OAA, the long range bound states (“dipole bound” states) are estimated with the large basis set aug-cc-pvtz.

Finally, the enthalpies and the Gibbs free energies of the fragmentation are calculated at ωB97x/aug-cc-pvtz level at 298 K and 400 K.

## 3. Results and Discussion

**Figure 2:**
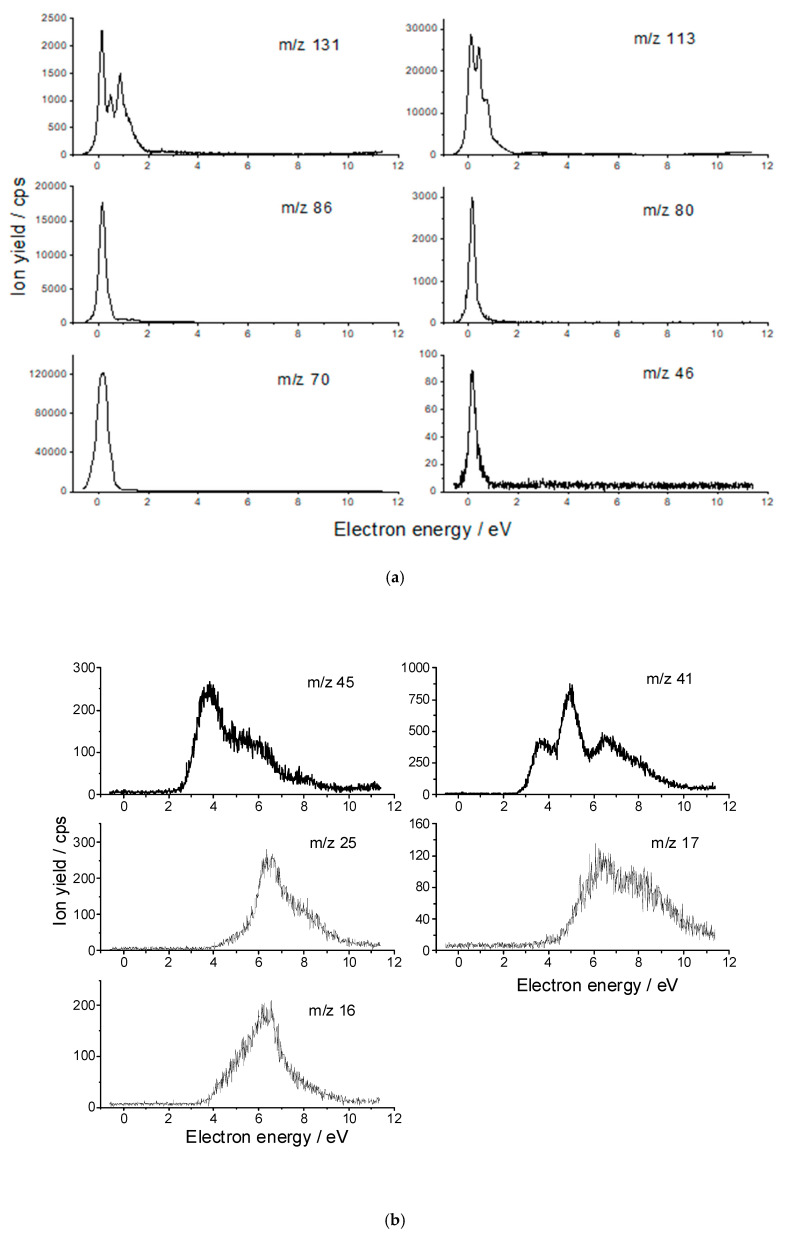
Anion yield functions of the oxaloacetic acid (**a**) fragment anions with m/z 131, 113, 86, 80, 70 and 46, and (**b**) fragment anions with m/z 45, 41, 25, 17, and 16.

**Figure 3:**
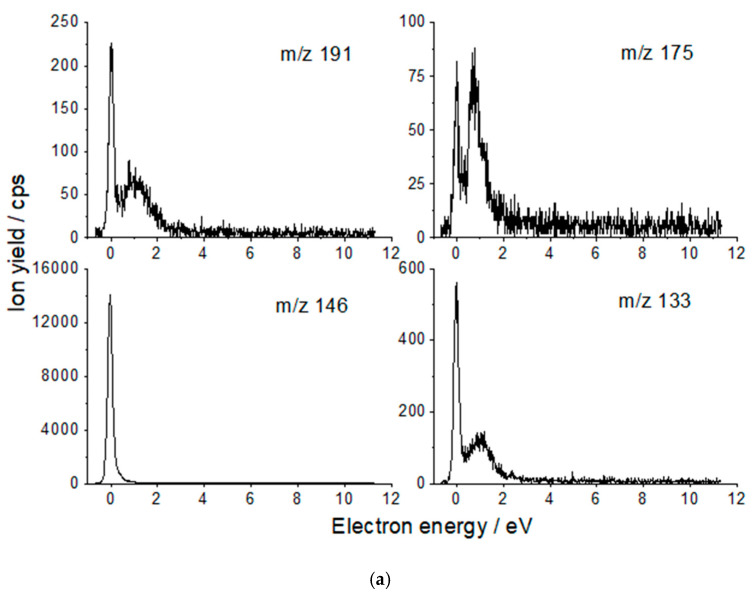

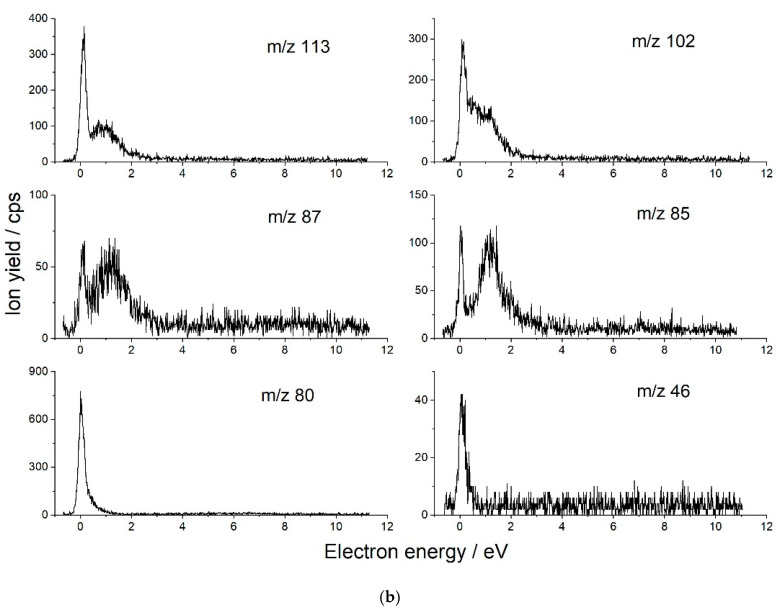
Anion yield functions of the citric acid: (**a**) fragment anions with m/z 191, 175, 146, and 133; (**b**) fragment anions with m/z 113, 102, 87, 85, 80 and 46.

**Figure 4:**
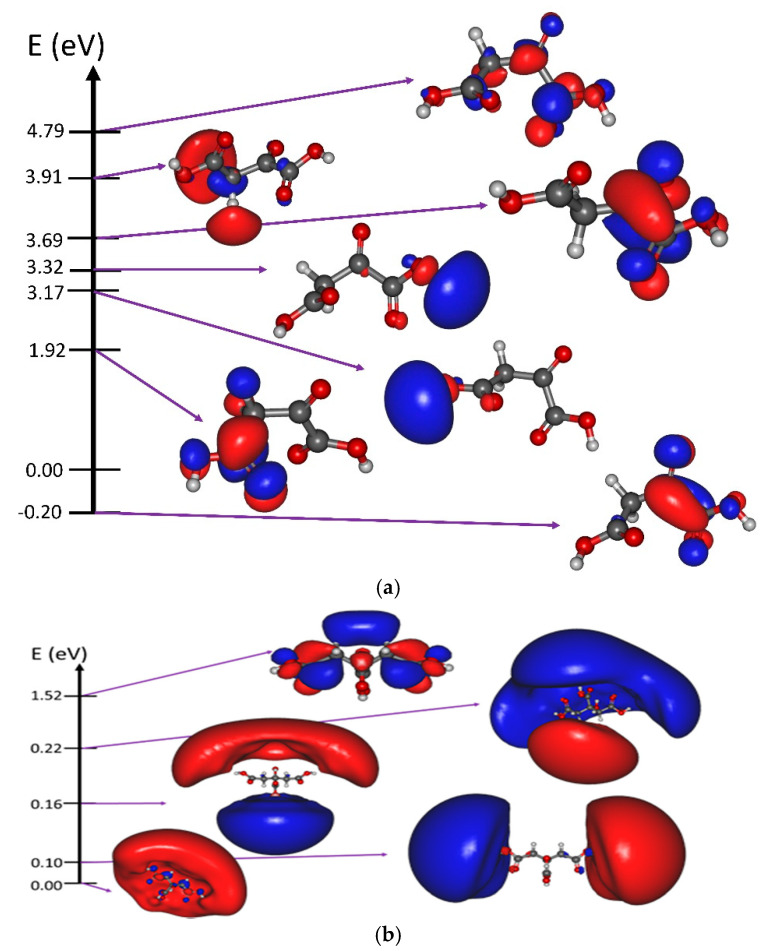
Calculated resonance energies (eV) and molecular orbitals associated to the attachment of the electron for (**a**) cis-enol oxaloacetic and (**b**) citric acids. Results obtained for the trans-enol OA are given in Figure S2. The cis-eno oxaloacetic acid was calculated at the ωB97x/cc-pvtz level at 0 K and the citric acid at the ωB97x/aug-cc-pvtz level of theory at 0 K since the presence of diffuse orbitals.

Figure 2 and Figure 3 present the recorded anion fragment yields, for OAA and CA, respectively, as a function of the energy of the incident electrons. Table 1 reports the resonance energies observed from these figures. The detected fragments were tentatively assessed by stoichiometry to the species listed in Table 1. The yield functions exhibit structures indicative of dissociative electron attachment (DEA), which is the most efficient mechanism for the molecular fragmentation at the electron energies below 10 eV. In DEA, the colliding electron is resonantly captured by the target molecule to form a transitory negative ion (TNI). If the lifetime of this TNI is longer than the dissociation time, the transitory negative ion dissociates into a negative fragment and one or more neutral counterparts [25]. Otherwise, the metastable parent anion may survive with a sufficiently long time to be detected mass spectrometrically as a parent anion, or the excess electron may auto-detach producing the neutral precursor molecule possibly in some excited state. The observed DEA signals in Figure 2 and Figure 3 are convolutions of the electron capture processes (or cross section) (as predicted by the calculated resonant states shown in Figure 4, Figure S2) and the survival probabilities (i.e., dissociation dynamics).

We will first consider the mechanism of the electron attachment for the formation of the TNI before considering the energetics for the production of the negative species and their neutral counterparts.

### 3.1. Resonant Electron Attachment and Formation of the Transitory Negative Anion

At electron energies below 10 eV, it is now established that the formation of the TNI may arise via three mechanisms, known as (a) the shape resonance, (b) the core-excited resonance [25] and (c) the dipole-bound mediated vibrational Feshbach resonance [26]. For the shape resonance, the incoming electron is trapped by the shape of the electron-molecule potential and occupies a usually unoccupied or a virtual molecular orbital. In the case of a core-excited resonance, the colliding electron excites one of the core-electrons into some electronic excited state, before being trapped by the positive core. Finally, the electron may transfer energy to some vibrational modes of the molecule, before being captured into a “dipole- or multipole-” bound state with a low binding energy, for which the excess electron occupies a very diffuse orbital outside the molecular frame [27]. If such a weakly bound state couples to some dissociative molecular valence orbital, the transitory anion undergoes dissociation [26]. All these mechanisms may arise in the 0–10 eV energy range [25,26,28,29]. Figure 4a,b exhibits the calculated resonance energies and the main molecular orbitals (MOs) associated with the attachment of the electron for OAA and CA, respectively. They represent the potential states into which the excess electron may be trapped to form the TNI.

For the OAA, the calculated MOs indicate that the excess electron occupies pure valence orbitals (Figure 4a) prior to the fragmentation of the TNI via competitive channels as shown in Figure 2. In the stable anion (energy of −0.20 eV in Figure 4a), the extra electron is located in a pi-type orbital at the COCOOH moiety of the cis-enol OAA. In contrast, the electron is located on the other moiety (CH_2_COOH group) of the molecule for excitation at 1.92 eV. Localization of the electron on peripheral hydroxyl groups occurs according to our calculations at 3.17 and 3.32 eV. The excitation energies of the trans-enol configuration (Figure S2) are somewhat similar to those of the cis-enol form. The calculated states presented in Figure 4a are associated with the shape resonance, except the resonance at 3.69 eV for which the attachment of the incoming electron with a spin alpha is concomitant with the HOMO-to-LUMO transition (spin beta).

The case of CA is interestingly different. The three lowest molecular orbitals exhibit a strong contribution of a very diffuse MO associated to a “multipole-”bound anion (due to the high values of the dipole and quadrupole moments and polarizability of the CA) with some contribution of valence molecular orbitals. Particularly at ~0 eV, the diffuse orbital encloses all three carboxyl groups (Figure 4b). The next diffuse MO localized at 0.1 eV is found to be close to the side carboxyl groups while at 0.16 eV, the excess electron density is localized near the central hydroxyl group. This diffuse “multipole-”bound state may then couple to the valence MOs, leading to the opening of very rich molecular dissociation decay channels, as observed in Figure 3. At the energy of 1.52 eV, the excess electron occupies pure valence MOs and the DEA is associated with the shape resonance.

### 3.2. Fragmentation of the Transitory Negative Anion

**Scheme 1:**
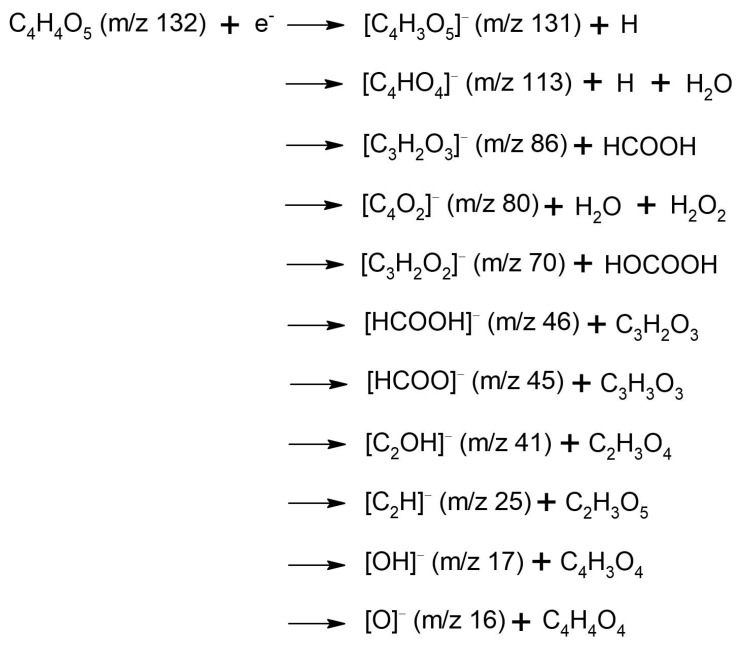
Fragmentation reactions of oxaloacetic acid upon interaction with low-energy electrons.

**Scheme 2:**
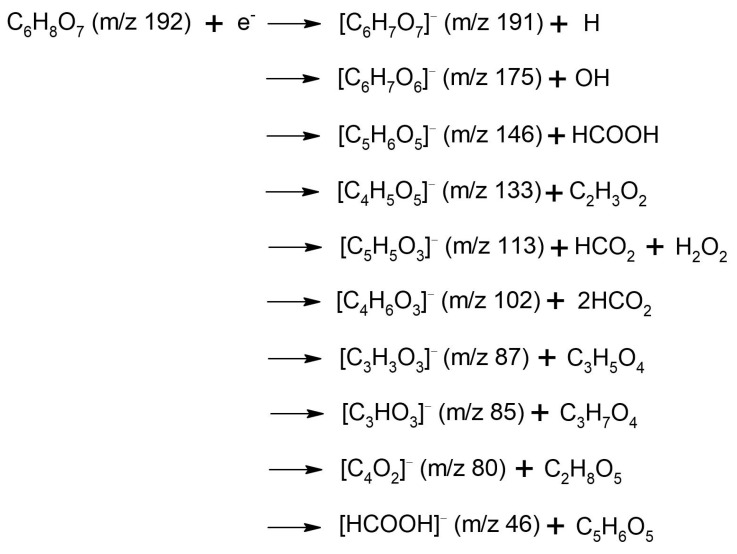
Fragmentation reactions of citric acid upon interaction with low-energy electrons.

In DEA, the fragmentation of the TNI produces a negative ion fragment, which is detected by mass spectrometry and one or more neutral counterpart(s). The different fragmentation pathways are depicted in Scheme 1 and Scheme 2. In a recent study by Pshenichnyuk et al., DEA to OAA was already reported, and a stable parent anion was observed [30]. The lifetime of the parent anion was estimated to be 25 μs, which is however too short to be observed in the presently used experimental setup. For molecules containing a hydroxyl or a carboxyl group, the dehydrogenation of the parent anion arises from the O-H cleavage near the energy threshold [31,32,33]. For the formic acid, this threshold has been reported at 1.15 eV [31]. The reaction enthalpy of electron-induced dehydrogenation of HCOOH can be described as a balance between the electron affinity of the HCOO (i.e., 3.17 eV [31]) and the HCOO-H bond dissociation energy (i.e., 4.47 eV [31]). In contrast, for the acetic acid, the pyruvic acid, the benzoic acid and the bromopyruvic acid this threshold is considerably below 1 eV [32,33,34,35]. In the present case, for both molecules, we observe the threshold for the molecular dehydrogenation near 0 eV (Figure 2a, m/z 131 and Figure 3a, m/z 191). We performed quantitative calculations on the energetics for the production of the hydrogen atom associated with the [M-H]- anion (M: OAA or CA), and also for the loss of the HCOOH moiety, which has been observed for both OAA and CA. The dissociation channel associated with the loss of a hydrogen atom has been observed for various molecular systems. Table 2 presents the calculated dissociation threshold for the formation of the dehydrogenated parent anion. For both molecules, it has been found that, at 0 K, the dissociation is not accessible near 0 eV, since the endothermicity of the reaction for the cis-enol structure of OAA and for CA (i.e., by 0.95 eV and 0.89 eV, respectively). The Gibbs free energies were also calculated at 400 K for both molecules. The dehydrogenation reaction from cis-enol OAA is found to be 0.19 eV, and the reaction is even more favorable for the trans-enol OAA since the dissociation threshold is calculated to be 0.05 eV. These results, for which the loss of the hydrogen atom arises at the carboxyl site, are in relatively good agreement with the experimental observations (Figure 2a, m/z 131). Furthermore, the near 0 eV resonance observed in the yield function of the [OAA-H]- anion suggests the contribution of the trans-enol form produced in the molecular beam. In the case of CA, the dissociation threshold at 400 K is calculated to be 0.17 eV’ again, in relatively good agreement with the experimental observation (Figure 3a, m/z 191). It is noteworthy that the loss of the hydrogen atom arises from the middle site carboxyl groups, while for the side carboxyl and the hydroxyl groups, the reaction energy is higher, i.e., 0.22 eV and 1 eV, respectively. At 298 K, the calculated Gibbs free energies of the [M-H]^−^ anions (M: CA or OAA) are found to be slightly higher in energy by about 0.10 eV (Table 2). Thus, we can predict that at room temperature some dissociation channels might be inactivated due to an increase of the thermodynamic threshold.

We have also examined the channel leading to the loss of either (H + COOH) or (HCOOH) for both molecules. For the citric acid, producing neutral H + COOH requires ~4.7 eV at 0K and ~3 eV at 400 K (Table 2). On the contrary, the cleavage of the central COOH and the H atom from the side carboxyl group concomitantly to the H + COOH recombination is exothermic by 0.80 eV (at 400 K). Since this channel has been observed near 0 eV (Figure 3a, m/z 146), we suggest the recombination to arise. The energetic scenario for the molecular dissociation is found to be similar for the oxaloacetic acid (Table 2, Figure 2a, m/z 86). At 298 K, the reactions mentioned above are found to be still exothermic (Table 2). In the case of OAA, the most intense peak observed at 0.17 eV is measured for the decay channel leading to the loss of a m/z 70 fragment (Figure 2a, m/z 70). The latter is likely to be associated with the loss of neutral (HOCOOH) to form the [OAA-HOCOOH]^−^ negative ion fragment. The reaction energies require 1.06 eV (1.01 eV) at 0 K, but only 0.13 eV (0.10 eV) at 400 K for the cis-enol (trans-enol) configuration (Table 2). At 298 K, the calculated Gibbs free energy is found to be 0.68 eV, i.e., 0.55 eV higher than that calculated at 400 K. Thus, this dissociation channel observed at 0.17 eV at 400 K might not be favorable at 298 K. It has to be noted that the carbonic acid, HOCOOH, is known to be an unstable species which rapidly undergoes dissociation to CO_2_ and H_2_O [36].

In addition to the loss of the hydrogen atom or the neutral formic acid, further decay channels lead to the dissociation of either a single (e.g., H_2_O_2_) or multiple (2xOH) neural product(s), as summarized in Table 1 and Scheme 1 and Scheme 2. They can arise through a very complex fragmentation scheme (e.g., C_4_O_2_^−^ anion fragment observed from the decomposition of the CA TNI), as a single or multiple steps [37].

The fragmentation of the OAA is mainly driven by the electrons with energies below 1eV (99%) as shown in Figure 2. We observe strong resonances already close to zero eV (i.e., 0.15 eV), and the decomposition is most likely driven by the formation of rather stable neutral molecules. Near threshold energies, we can estimate the branching ratio for the formation of the [C_4_H_3_O_5_]^−^, [C_4_HO_4_]^−^, [C_3_H_2_O_3_]^−^, [C_4_O_2_]^−^, [OAA-HOCOOH]^−^, and [HCOOH]^−^ anion fragments and their associated counterpart to be 3.8%, 13.6%, 4.0%, 1.9%, 76,7% and 0.05%, respectively. These branching ratios reflect the dynamics of the dissociation of the TNI at 400 K. Although the loss of the (HOCOOH) neutral species resulting in the anionic fragment at m/z 70 is by far the most dominant dissociation channel, the production of other neutral radicals such as H_2_O_2_ (or 2 OH) and ballistic hydrogen atoms, represents almost 23% of the decay channels. These radicals may then play a role in the modification of the medium under irradiation when containing OAA molecules. In the case of CA, the molecular fragmentation is induced by electrons with energies below 1 eV, for which 84% arise from the near 0 eV electrons dominated by the loss of the (HCOOH) neutral(s) associated with m/z 146 (Figure 3a), and 16% from ~1 eV electrons (Figure 3b).

## 4. Conclusions

The present work reports a comparative study of dissociative electron attachment to oxaloacetic and citric acid. The two molecular systems show fragmentation of the transitory negative ion by near 0 eV electron attachment to the neutral parent molecule. However, the process is completely different in both molecules. Indeed, for the oxaloacetic acid, the dissociation operates via the attachment of the excess electron into some valence molecular orbital while for the citric acid, it is very likely to arise initially from the formation of a “dipole-multipole-”bound anion as a gateway for the dissociation of the covalent TNI. The decomposition of both metabolites by low-energy electrons could be associated with a disturbance of a cell’s metabolism and therefore the results found here may represent important pathways of radiation induced cell damage.

Nevertheless, in the condensed phase situation the molecules are surrounded by some environment (e.g., H_2_O for biological type of environment). In this case it is well known that the electron attachment process may change [38,39]. For instance, it has been demonstrated that the dipole bound character may disappear for the benefit of a valence bound anion, when the molecule is solvated by a couple of water molecules from the environment [40]. Thus, the citric acid might not be sensitive to the electron attacks in contrast to the oxaloacetic acid in condensed media. In contrast, the decomposition of the oxaloacetic acid by low energy ballistic electrons may produce reactive species such as hydrogen peroxide contributing to the alteration of nucleobases [41], the formic acid involved in the glycosidic bond cleavage or the hydrolysis of DNA [42] or the CO_2_ as a mediating agent for DNA damage [43].

## Figures and Tables

**Table 1:** List of the detected m/z negative species created by DEA to oxaloacetic acid (OAA) (left) and to citric acid (CA) (right). The resonance energies are obtained from Figure 2 and Figure 3. The suggested associated radical to the negative fragments and its formation energy are discussed in the text. (* weak peak). In blue the same formula is given in terms of neutral loss channels.

Anion Fragment from OAA m/z	Resonance Energies (eV)	Anion Fragment from CA m/z	Resonance Energies (eV)
131 [C_4_H_3_O_5_]^−^ [OAA-H]^−^	0.14 0.48 0.88 2.78 *	191 [C_6_H_7_O_7_]^−^ [CA-H]^−^	0.01 0.99
113 [C_4_HO_4_]^−^ [OAA-H_2_O-H]^−^	0.15 0.44 0.71 1.13 2.75 * 4.8 *	175 [C_6_H_7_O_6_]^−^ [CA-OH]^−^	0.0 0.85
86 [C_3_H_2_O_3_]^−^ [OAA-HCOOH]^−^	0.14 1.3 * 3.1 * 6.4 *	146 [C_5_H_6_O_5_]^−^ [CA-HCOOH]^−^	0.0
80 [C_4_O_2_]^−^ [OAA-H_2_O-H_2_O_2_]^−^	0.16 0.56 1.4 *	133 [C_4_H_5_O_5_]^−^ [CA-C_2_H_3_O_2_]^−^	0.0 0.94
70 [C_2_H_2_O_2_]^−^ [OAA-HOCOOH]^−^	0.17 0.53 * 1.3 2.83 * 4.4 *	113 [C_5_H_5_O_3_]^−^ [CA-H_2_O_2_-HCO_2_]^−^	0.12 0.89
46 [HCOOH]^−^	0.17	102 [C_4_H_6_O_3_]^−^ [CA-2(HCO_2_)]^−^	0.11 0.41 0.98
45 [HCOO]^−^	3.7 5.0 6.3 7.0	87 [C_3_H_3_O_3_]^−^	0.09 1.22
41 [C_2_OH]^−^	3.7 4.95 6.6 ~8.0	85 [C_3_HO_3_]^−^	0.04 1.20
25 [C_2_H]^−^	6.5 8.0	80 [C_4_O_2,_]^−^	0.05
17 [OH]^−^	5.3(4) * 6.3 8.1	46 [HCOOH]^−^	0.09
16 [O]^−^	5.2 6.3 7.0		

**Table 2:** Calculated fragmentation thresholds (DFT level of theory) of cis-enol oxaloacetic (OA) and citric (CA) acids: enthalphy (0 K) and Gibbs free energy at the ωB97x/aug-cc-pvtz level at 400 K. Energies in eV. * Values for trans-enol OAA are given in parentheses. The values in red are Gibbs free energies calculated at 298 K.

	M = OAA *		M = CA	
	Enthalpy	Gibbs Free Energy	Enthalpy	Gibbs Free Energy
**[M-H]^−^**	0.95 (0.84)	0.19 (0.05) 0.31 (0.17)	0.89	0.17 0.27
**[M-HCOO-H]^−^**	4.78 (4.73)	3.05 (3.02) 3.38 (3.34)	4.72	2.98 3.31
**[M-HCOOH]^−^**	0.22 (0.17)	−0.73 (−0.76) −0.53 (−0.57)	0.16	−0.80 −0.60
**[M-HCOO-OH]^−^**	1.06 (1.01)	0.13 (0.10) 0.68 (0.66)		

## Data Availability

Data is available from the corresponding authors upon request.

## Supplementary Materials

The following are available online at https://www.mdpi.com/article/10.3390/ijms22147676/s1, Figure S1: Optimized geometry of the trans-enol oxaloacetic acid at 0 K, Figure S2: Calculated resonance energies (eV) and molecular orbitals associated with the attachment of the electron for the trans-enol oxaloacetic acid, calculated at the ωB97x/cc-pvtz level of theory at 0 K.
