# Supplementary material for: Experimental and Theoretical Studies of Dissociative Electron Attachment to Metabolites Oxaloacetic and Citric Acids
Source: Int J Mol Sci. 2021 Jul 18;22(14):7676. doi: 10.3390/ijms22147676 (PMC8303309; doi:10.3390/ijms22147676)

SUPPLEMENTARY INFORMATION

Figure S1: Optimized geometry of the trans-enol oxaloacetic acid

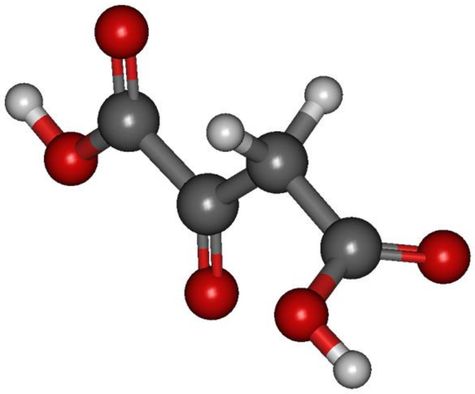

Figure S2: Calculated resonance energies (eV) and molecular orbitals associated with the attachment of the electron for the trans-enol oxaloacetic acid, calculated at the ωB97x/cc-pvtz level of theory at 0 K.

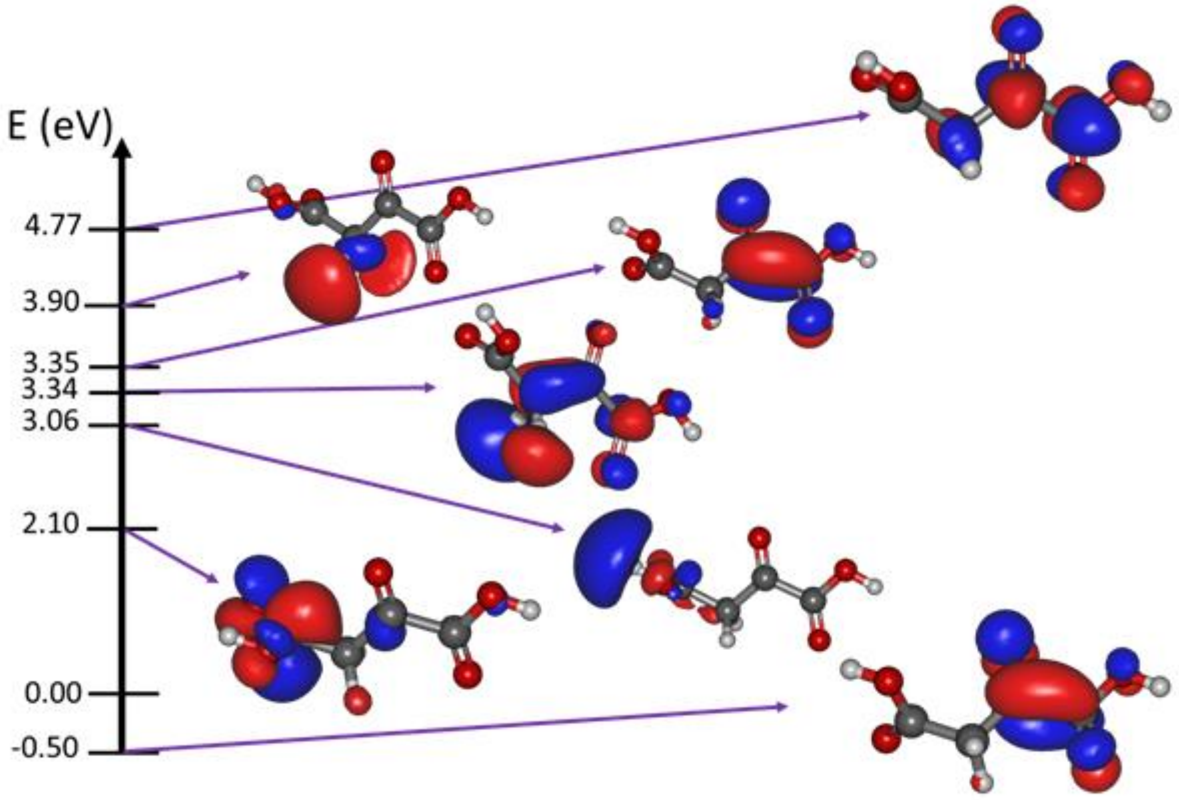

Supplement: Supplementary file 1 [file ijms-22-07676-s001.zip › ijms-1270893-supplementary.pdf]
